# Could a hand-held, visual electrophysiology device theoretically reduce diagnostic waiting times for complex eye conditions in the NHS? A Discrete Event Simulation (DES) modelling study

**DOI:** 10.1186/s12913-025-12551-w

**Published:** 2025-03-28

**Authors:** Steffen Bayer, Daniel Garillo, Marion Penn, Maria Chorozoglou, Sally Brailsford, Eloise Keeling, Fatima Shawkat, Perry Carter, Helena Lee, Jay E. Self

**Affiliations:** 1 Clinical and Experimental Sciences, Faculty of Medicine, University of Southampton, Southampton, UK; 2 Ophthalmology Department, University Hospital Southampton, Southampton, UK; 3 Southampton Health Technology Assessments Centre, Faculty of Medicine, University of Southampton, Southampton, UK; 4 Southampton Business School, University of Southampton, Southampton, UK

**Keywords:** Ophthalmology, Electrophysiology, Electoretinogram, DES modelling, RETeval^TM^

## Abstract

**Background/objectives:**

Visual Electro-Diagnostic Testing (EDTs) are a highly specialised service in the NHS. The high cost of tests and a paucity of trained visual electrophysiologists has resulted in very few services across the UK and, when combined with increasing patient backlogs, has caused significant travel burden and variable waiting times. Here, we study the potential for impact on patients and services by adding a screening step to traditional referral pathways using an Electroretinogram (ERG) test from a relatively inexpensive, portable, hand-held EDT device; the RETeval^®^ (LKC technologies, Gaithersburg, MD, USA).

**Subjects/methods:**

We model a large regional-referral EDT service using Discrete Event Simulation (DES) modelling based on retrospective patient data and published best evidence for the device. We evaluate the potential impact that adding the screening step in referral pathways could have on patient waiting times should the device prove to be safe and useable in clinical practice.

**Results:**

We demonstrate that should the RETeval^®^ ERG be safe and useable in real-world clinical practice, it has the potential to significantly reduce patient waiting times by avoiding lab-based EDT assessment for up to 45% of patients. We also show that the impact on services and patients is likely to be resilient to realistic changes in referral numbers, sensitivity/specificity of the device and changes in clinical capacity.

**Conclusions:**

This work demonstrates that a RETeval^®^ ERG screening step, performed at the point of referral, has the potential to result in significantly reduced EDT waiting lists through fewer patients requiring lab-based EDT assessment and that DES modelling is a useful tool in making this assessment. However, many questions remain about using the device in the real-world setting for this purpose. Future studies are needed to assess its sensitivity/specificity, test/retest variability, changes in referral patterns due to the device, useability, acceptability to patients and importantly, the consequences of screening errors. Our work, using only retrospective data and a DES model, shows that using the device as an ERG screening tool warrants further investigation due to the potential impact on both patients and clinical services.

## Introduction

Like many other NHS services, Ophthalmology in the UK is under significant strain. This is partly a result of the COVID-19 pandemic but also due to both an ageing population and advances in treatment which necessitate more investigations per patient. Indeed, Ophthalmology is the busiest hospital outpatient speciality and makes up almost 10% of the current 7.5 M NHS backlog (www.england.nhs.uk/statistics/statistical-work-areas/rtt-waiting-times/. NHS England 2023).

Some Ophthalmology services are highly specialised and are only provided by a few centres in the UK, including visual Electro-Diagnostic Testing (EDT). This specialised service is provided by senior clinical scientists. The most common investigations include Electroretinography (ERG) which assesses the function of the retina and Visual Evoked Potentials (VEP) - a measure of the electrophysiological response of the brain to visual stimuli. These techniques are performed to international standards (ISCEV) [1] or amended, less stringent protocols to take account for less compliant patients or children such as the ‘GOSH’ paediatric protocol [2, 3]) in bespoke clinical laboratories. They are a powerful non-invasive diagnostic tool in both ophthalmology and neurology [4, 5]. However, due to the rarity of trained individuals, the high cost of the testing equipment and the necessity for a large laboratory setup, dedicated ophthalmic EDT services are only provided by a handful of centres and patients often travel long distances and wait for a long time, although waiting times vary from centre to centre. Therefore, the backlogs and scarcity of these expensive services comes at a cost to both patients, their families, and to the NHS.

Within the visual electrophysiology community, there has long been a consensus that the service delivery model will need to change to retain complex, detailed assessments at specialist centres, while developing alternative, accessible ways to deliver these essential tests to the huge numbers of people who cannot yet access their benefit [5]. The tests need to be quicker and easier, diagnostically more robust, less onerous for patients, and more widely available [5]. Several innovations in the field are being explored by our group and others including the use of virtual reality, wireless electrodes, advanced signal processing, eye-tracking machine-learning, harmonised reference data and more [6–8]. However, at present, access to EDTs is still a major bottleneck in many diagnostic and monitoring pathways and this represents a health inequality not least because of the geographical scarcity [5].

In recent years, a hand-held device (RETeval^®^) has been developed which, at a fraction of the cost of the full laboratory setup, can perform some of the more common electrophysiology assessments (Flash VEP and pattern ERG). *Importantly*,* it has the potential to be used by a relatively untrained individual*,* in a non-dedicated environment*. Some studies by our group and others [9–11] have shown that the device performs well in defined patient groups when compared with the lab-based evaluation, raising the question of whether it could be used as a screening tool in the hands of referring clinicians to reduce the pressure on EDT services by removing patients with normal findings at the point of referral. Furthermore, the literature broadly supports the efficacy of RETeval^®^ generated ERGs in a broad age range [9–12], the speed of the test and lack of need for pupil dilation [12], the sensitivity and specificity for identifying a range of ophthalmic conditions [9–18] as well as the ease of use in non-expert electrophysiologists hands and outside of the diagnostic laboratory setting [12]. However, most of these studies have included small patient numbers and been completed in research settings by expert electrophysiologists using the RETeval^®^ device only in specific patient groups (types of referrals) and its agreement with standard laboratory-based EDT assessments has been reported variably in the literature according to these factors. Additionally, the impact of using the RETeval^®^ ERG as a screening tool for retinal disorders in complex clinical referral networks has not yet been explored for safety or useability. In this project, we sought to study whether using the device for this purpose offers enough theoretical promise (in terms of removing patients from the referral pathway and reducing waiting lists, to warrant further clinical research and de-risk future research investment.

## Methods

### Retrospective clinical data collection

This study performed as part of a clinical service review and registered with the University Hospital Southampton Health Service Review (HSR) committee. Retrospective, anonymised data were collected from a single large tertiary referral electrodiagnostic service for the pre-COVID19 period of Sept-Nov 2019. This period was used to avoid the impact of the pandemic on both patient referrals and clinical appointment availability and reflect the typical clinical picture by avoiding the significant impact on both caused the pandemic. Data were collected for every patient seen within this 3-month period using in-house electronic patient records. All patients had received full laboratory standard EDT assessments either using the ISCEV standard protocol (adults) [1] or GOSH amended protocol (children and non-compliant adults) [2, 3]). Three clinical team members extracted data including patient age, referral date, EDT investigation date, referral reason, and whether the ISCEV-standard ERG was reported as normal or abnormal. The referral reasons were categorised into one of 4 groups to aid data analysis (see Table 1) and all data was anonymised.

**Table 1:** Patient referral categories used for DES modelling

**Group A** Retinal symptoms or findings (e.g. nyctalopia or retinal pigmentation)
**Group B** Retinal disease risk due to non-ocular reason (e.g. systemic diagnosis which is associated with retinal disease or family history of retinal disease)
**Group C** Retinal disease risk due to non-retinal, ophthalmic findings (e.g. unexplained high myopia in a child or unexplained reduced vision)
**Group D **Referral primarily for Visual evoked potential (VEP) evaluation and therefore not suitable for screening with a hand-held ERG.

Patients in Group D (those referred primarily for visual evoked potential (VEP) evaluation and therefore not suitable for screening with a hand-held ERG) were included in the model (despite not passing through the RETeval^®^ screening step pathway) as these patients would still be part of the overall patient burden in the queue for lab-based EDT evaluation. The model also included groups A-C and in experiment 5 we study each of these 3 groups separately. Patients referred for screening or monitoring (e.g. hydroxychloroquine screening or for Birdshot chorioretinopathy) are included in Group B for their first evaluation only and for subsequent appointments are not included in the screening model.

The backlog of patients on the waiting list for EDT at University Hospital Southampton used for the model was 228 (taken from data at Nov 30th 2019) and the average number of appointment slots for EDTs per week (capacity) was 12.

### Discrete event simulation model development

Simulation was used to model the impact of the introduction of a screening step with a handheld ERG test. A significant advantage of simulation modelling as a step before implementation or experimentation in the real system is that it allows the exploration of a variety of changes to the systems quickly and efficiently with no patient risk. Simulation has been widely used to effectively model and affect change in a variety of healthcare scenarios [19–21]. Work addressing waiting times for healthcare services frequently employs discrete event simulation (DES) because this simulation approach also allows capturing real-world variability between individual patients and does not rely on averages. Discrete Event Simulation (DES) conceptualises healthcare systems as a series of queues and activities, with patients moving through the system waiting in queues between activities.

The work using DES for analysing waiting times in healthcare falls into two categories. Much work focuses on the waiting time of the patient at a clinic (measured in minutes or hours), while other work focuses on the waiting for an appointment or a scheduled admission (measured in days or weeks).

Many studies in the first category examine emergency department operations, often motivated by targets to see patients within specific time frames [22–26]. Similar work has also been done for scheduled outpatient appointments [27], waiting times in primary health care centers [28], ophthalmic outpatient clinics [29] or the impact of service integration on patient time for HIV and outpatient services [30].

Work in the second category is less frequent and uses DES modelling to understand and improve the longer waiting times for scheduled admissions and appointments. Simulation studies in this category include work on whole hospital operations and achieving waiting time targets for outpatient appointments and elective admissions [31], a study on strategies to meet waiting time targets for a rheumatology clinic [32] or work on the impact of adding resources to an obesity care service.

The work presented in this paper falls into the second category: the focus was to reduce the waiting times for an appointment for EDT which presently is many weeks. A DES model was therefore developed to study the referral pathway for EDT evaluation including steps from initial referral, to results of the EDT being available to the referring clinician. Patients were either pre-existing as a backlog, referred to the EDT service from local referring centres, or referred from in-house clinics. The model was built to reflect each step in the pathway and the retrospective clinical data inputted.

**Fig. 1:**
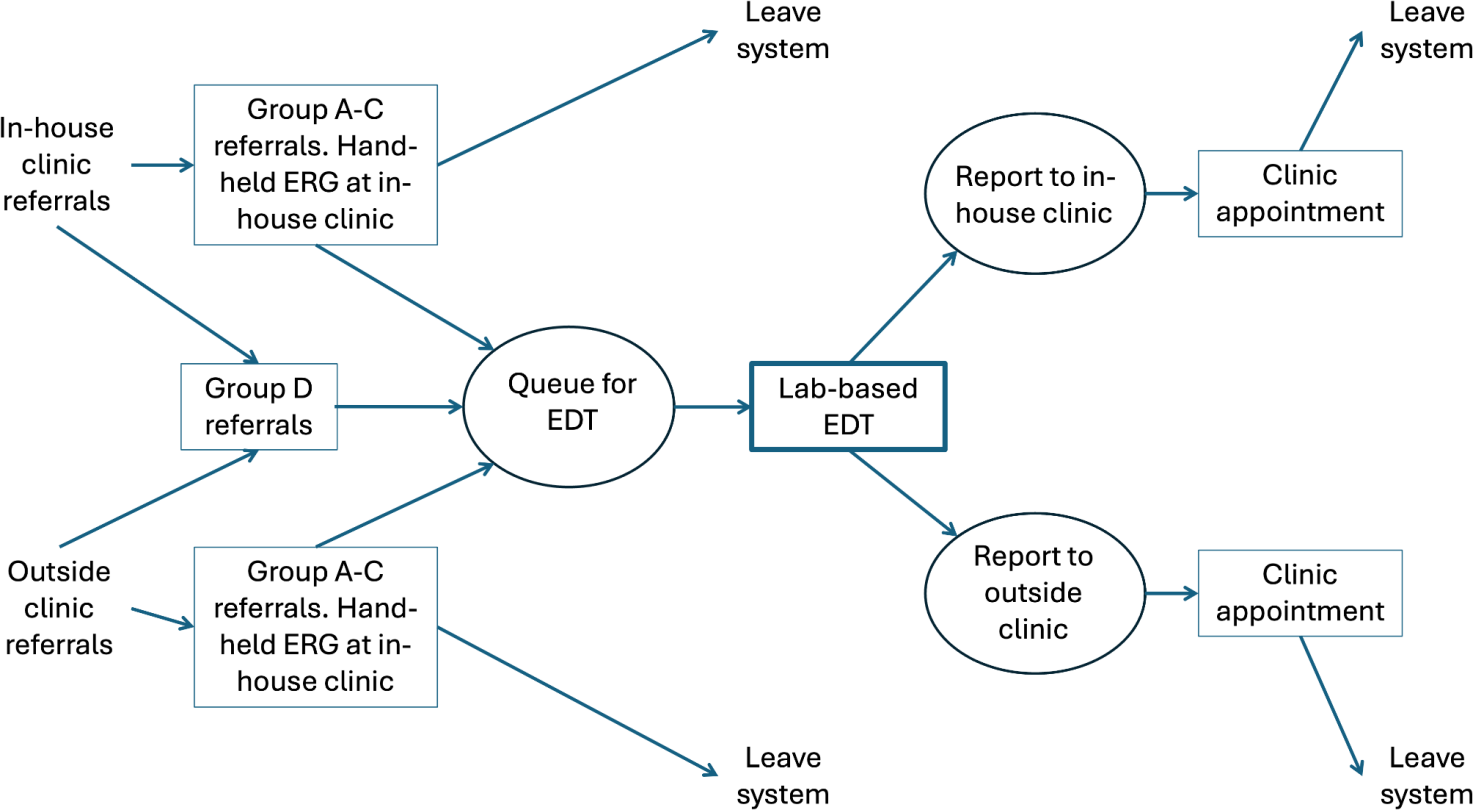
Current EDT pathway model with additional RETeval^®^ ERG screening step. (RETeval^®^ model). ‘EDT’ refers to Electrophysiology assessments and ‘ERG’ refers to Electroretinogram

The section of the ophthalmology pathway that could be influenced by using the RETeval^®^ ERG screening step was established through discussion with the clinicians in the team. This was used to produce the flow diagram shown in Fig. 1, which was then modelled using the DES software Simul8 (https://www.simul8.com/). The purpose of the model was to study the primary question: what would be the impact on the diagnostic pathway of adding a RETeval^®^ ERG screening step at the point of referral? For the base case with no RETeval^®^ ERG screening step, the model is set so that all patients flow straight through to the queue for EDT, for other scenarios the flows at this stage are set to reflect the specific experimental scenarios. For all scenarios except that looking directly at the effect of sensitivity and specificity of the RETeval generated ERG, it was necessary to assume a standard sensitivity and specificity threshold for the device in order to permit individual experiments. A threshold of 95% for both was chosen based on a broad interpretation of the existing literature described above and our own published experience with the device [9–12, 33]. However, it is appreciated that this requires further prospective study as it may vary in real-world clinical practice across larger networks.

The model was populated using the above-mentioned retrospective clinical data (see also Table 4 in Appendix) and the base case (no changes to the current system) was validated using the clinician’s expert opinion. Patients currently on the waiting list were added to the wait for EDT at the start of each run. The arrival rates of new referrals, and the proportions in each category, are based on the retrospective data. As the category D patients are not being considered for RETeval^®^ ERG screening, but would influence the number of lab-based EDT appointments used and therefore affect waiting times, they arrive directly into the queue for EDT. To encompass the variations in patient arrivals and treatment times, each scenario was replicated 2000 times. The key output measures were the waiting time for EDT and the proportions of patients correctly identified by screening. A range of different scenarios were considered. For each scenario, the model was run twice; first for five years starting at time zero with the current backlog of patients, and then for six years with a 5-year ‘warm up’, i.e. discarding the data from years 1–5 and considering only year 6, to see whether the waiting time had stabilised or was continuing to grow.

## Results

### Retrospective clinical data results

A search on the dedicated in-house EDT EPR identified 261 consecutive cases undergoing EDT assessment from Sept-Nov 2019 of which 195 had full data. Of these, 131 cases fell into referral Groups A-C and 64 into Group D (please see Table 2).

**Table 2:** Retrospective data collection. Consecutive patients from a single large EDT service. Those with missing data excluded

	*n* for 3 months with full data (collected data)	Expected *n* for 1 year (calculated data)	Number of patients with abnormal ERG identified by gold-standard ERG in 3 months (collected data)	Expected Number of patients with abnormal ERG identified by gold-standard ERG in 1 year (calculated data)	% of patients with a normal gold-standard ERG (collected data)	Average delay in days between referral and EDT appointment (collected data)
**Group A**	77	308	23	92	70%	93
**Group B**	14	56	4	16	71%	88
**Group C**	40	160	5	20	87%	101
**Group D**	64	256	N/A	N/A	N/A	95
***Totals***	**195**	**780**	N/A	N/A	N/A	**95**

*N/A* not appliicable

Only those patients with full data were used for input into the DES model, as many of those with missing data were duplicate appointments or EPR errors. However, it is possible that some patients who did impact on the service have been excluded and the referral rates used in the model are underestimates. Therefore, it is possible that in reality, patient waiting times might be even longer than the results suggest.

Furthermore, the current clinical backlog in our service of 228 was used for the model although it is appreciated that backlogs vary at different times and between different referral networks/centres.

### Discrete Event Simulation (DES) model results

In this section we describe the results of 5 experiments using the core data described above to answer different questions about the impact of a screening step in referral pathways for visual electrodiagnostics. Table 3 includes results from all 5 experiments which are then discussed in order.

**Table 3:**
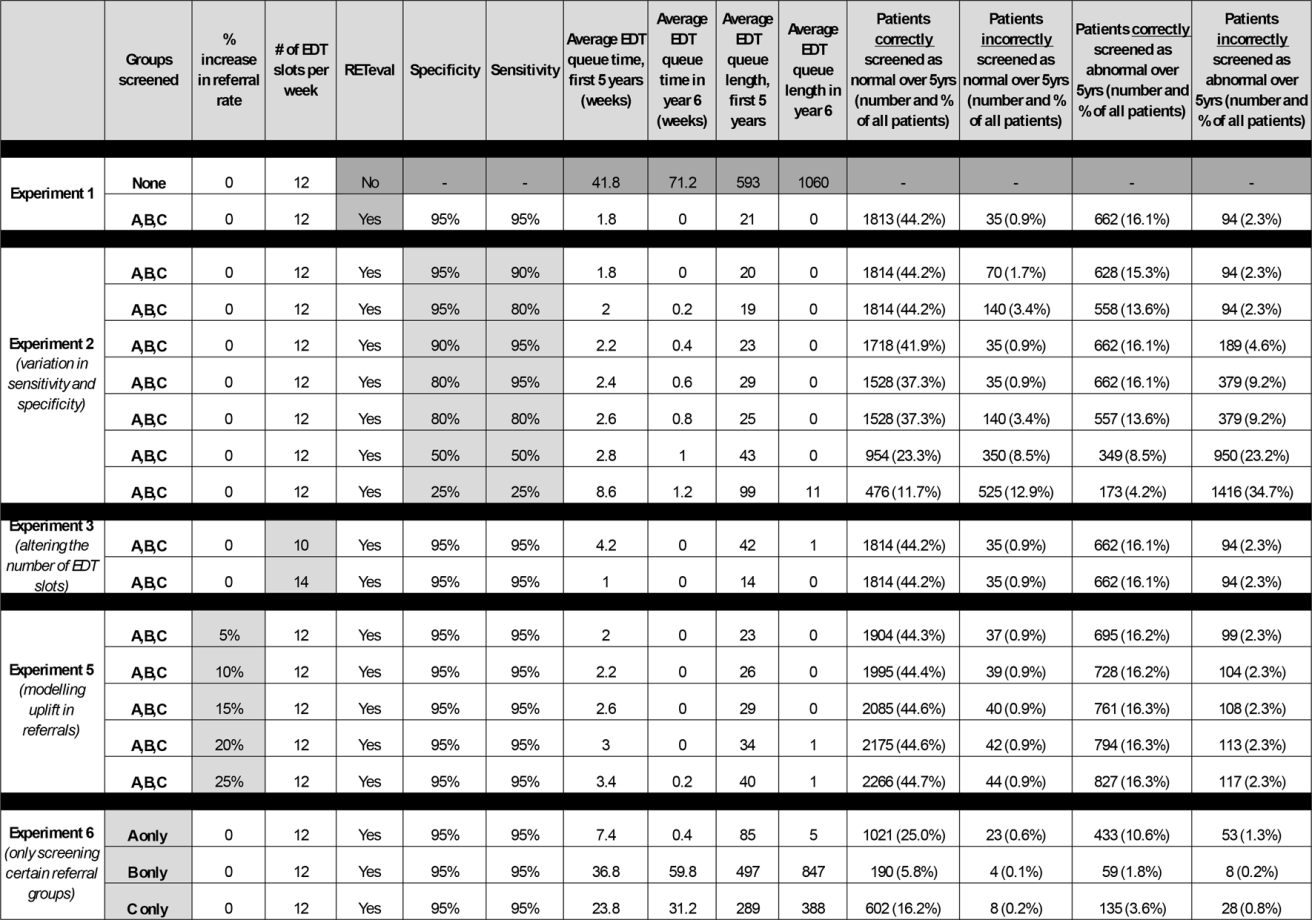
Discrete event simulation (DES) model experimentation results. The stated percentages in the final 4 columns are as a percentage of the overall number of patients referred for lab-based electrophysiology. Therefore, the denominator value includes group D patients, i.e. Those for which a RETeval^®^ ERG would not be applicable as their referral is primarily for a visual evoked potential (VEP) test but do form part of the lab-based electrophysiology clinical burden

Shaded areas represent those elements under study in each experiment

### What is the effect on waiting lists for EDT service of adding the *RETeval*^®^ ERG screening step at the point of referral, assuming 95% sensitivity/specificity? (experiment 1, Table 3)

Here we compared the *status quo* pathway vs. the current pathway with the addition of a RETeval^®^ screening step. We assumed a sensitivity and specificity of 95% for the screening step based on the best available evidence from the literature in published work by our group and others [9–18] although it is noted that research is required to study this in a similar population of patients for more accuracy in the future. For the screening option, the average wait in years 1–5 is 2 weeks and there is no queue at all in year 6. However, in the *status quo* scenario, the average wait in years 1–5 is 42 weeks and increases significantly to 71 weeks in year 6.

Notably, whilst the *RETeval*^®^ screening step scenario reduces the number of lab-based EDT assessments required in the first 5 years from 4100 to 2252, 35 of the 2604 cases undergoing a *RETeval*^®^ screening step (0.9% of patients) would be incorrectly screened out of the service and so represent false negative screening which poses a clinical risk.

### What is the effect of varying the sensitivity and specificity of the *RETeval*^®^ ERG screening step on the new pathway model? (experiment 2, Table 3)

Our model thus far has assumed a sensitivity and specificity for the *RETeval*^®^ ERG of 95%. This is based on current evidence but data is lacking on whether this reflects reality and would be key to clinical use and safety. To study the effect of sensitivity/specificity, we ran the screening step scenario using a range of different sensitivity and specificity thresholds for the ERG device. In all cases, the average waiting time in years 1–5 is much smaller than the *status quo* and (with the exception of the extreme case when the sensitivity and specificity are both only 25% which would make the device unusable anyway) the average waiting time has fallen to zero in year 6.

By reducing the sensitivity of the screening step from 95 to 90%, the number of patients incorrectly screened out of the pathway doubles from 35 to 70.

By reducing the specificity of the screening step from 95 to 90%, the number of patients incorrectly screened out of the pathway is unchanged but the number who are incorrectly screened as abnormal doubles (from 94 to 189).

The scenarios with sensitivity and specificity levels between 25-80% illustrate that even if the test has extremely low sensitivity and specificity, the effect on reducing the number of people in referral pathways are still strong. However, safety is paramount for any screening tool and the number of incorrectly screened patients increases rapidly as the sensitivity reduces. This highlights the need for additional evidence to identify the sensitivity/specificity of the screening ERG in clinical practice and the real-world clinical consequences of incorrect screening.

### What is the effect of varying the number of laboratory EDT slots on the new *RETeval*^®^ ERG screening step model? (experiment 3, Table 3)

We explored the effect of altering the number of full EDT slots per week from the default 12 used in all other scenarios to either 10 or 14, assuming a sensitivity and specificity of 95% for the *RETeval*^®^ ERG again for the reasons stated above.

Although the number of EDT slots per week affects the waiting time, in all three scenarios, the RETeval screening step produces waiting times that are significantly lower than the status quo (no screening) scenario. By year 6, even with only 10 slots per week the average waiting time is still zero compared with 71weeks in the status quo, 12 slots per week scenario. This suggests that the effect of altering the capacity for lab-based EDT slots is dwarfed by the effect of the new screening step.

### What is the potential effect of increasing the number of patient referrals on the new *RETeval*^®^ ERG screening model (increased demand)? (experiment 4, Table 3)

The number of patients referred for EDT evaluation is likely to vary across the UK but also, due to increasing numbers of patients entering ophthalmic services across the NHS. It is also conceivable that implementation of the *RETeval*^®^ ERG screening scenario in the clinics may cause a change in clinicians’ practice resulting in more patients undergoing EDT evaluation due to the availability of the device and reduced waiting times for lab-based EDTs. Here we study the resilience of the *RETeval*^®^ ERG screening scenario to an increase in patient numbers regardless of cause. We again assumed a sensitivity and specificity of 95% and modelled the effect of increasing the number of referrals into the system according to the current ratio patients in each referral group.

As seen in Table 3, waiting times increase as the referral rates increase as expected, but the system seems very resilient. Indeed, even with the maximum modelled increase of 25%, the average waiting time in year 6 is still zero, compared with 71 weeks in the *status quo* pathway without any increase in referrals.

### What is the effect on the model if the *RETeval*^®^ ERG screening step is only implemented in specific patient subgroups according to referral type? (experiment 5, Table 3)

In our modelled scenarios so far, we have combined the effect of a screening step on all patients regardless of referral type. However, it can be seen from Table 2 that the percentage of abnormal ERGs differ according to the reason for referral and the number of patients in each referral group also differs. It is therefore possible that the addition of a RETeval^®^ ERG screening step might be most impactful when used in a specific group of patients. Here we model the effect of only screening either referral groups A, B, or C alone whilst measuring the impact on whole patient cohort. Again, we assumed a sensitivity and specificity of 95% (see Table 3).

As expected, employing the screening step only for a subset of patients reduces the overall effect on patients entering the referral pathway and thus waiting times. In particular, screening the two smaller groups (B and C) only has relatively little impact and results in long waits in years 1–5 (36 and 24 weeks respectively) and in year 6 (60 and 31 weeks respectively). However, it is notable that screening the largest patient group (group A) still has a large effect and all scenarios in which fewer patients are screened accordingly result in fewer false negative and false positive screening errors.

## Discussion

We have sought to explore the value of future clinical research to evaluate the potential of introducing the *RETeval*^®^* ERG* as an additional screening step when patients enter a referral pathway for visual electrodiagnostics (EDTs). Our DES model has been built upon consecutive patient data from a 3-month pre-COVID period and current evidence in the published literature regarding the capabilities of the *RETeval*^®^ ERG. The simulation has allowed us to model the potential impact on both patients and the NHS service in broad terms including how the screening step is predicted to reduce and remove a current backlog and reduce patient waiting times. Although our data only models one regional referral service and current backlogs and referral numbers are likely to vary from centre to centre, it is likely that other regional services would respond in similar ways. Our data have shown that twice as many patients are referred to the EDT service predominantly for an ERG evaluation than are referred primarily for a VEP although again, this may vary according to patient details such as age and from service to service. The current best evidence from the published literature supports the efficacy of the RETeval^®^ ERG in identifying retinal abnormalities and that this is applicable to a wide range of age groups. In order to test different hypotheses, we had to assume a standard sensitivity and specificity for the device and chose 95% for all experiments except those looking directly at the effect of altering these values. However, these thresholds need to be tested in real-world clinical use in order to define the true values if the device should be used as a screening tool. Some literature and our own experience suggests that a potential advantage of the device is that it could be used by clinicians with minimal training. However, again, how much training is required and the drop-off in useability needs evaluation. We have shown that that the impact on waiting times is dependent on several factors including the sensitivity and specificity of the tool and the case mix which is likely to vary between referral networks. Nonetheless, the impact of these variables seems far smaller than the enormous potential impact of the additional screening step. For example, even if the tool had far lower sensitivity and specificity (or compliance rate) than the current literature would predict (for example 80% for both), due to the sheer number of cases with normal ERG results, average waiting times are greatly reduced, and by year 6 there is no waiting list at all. An important consideration is that besides the overall maximum sensitivity and specificity of the device, the criteria used to define a test as normal, borderline, or abnormal could be altered within the software of the screening tool. There will be a limit on how high the sensitivity and specificity can be, dictated by the technology, and a limit on useability in the real-world setting and these factors would also need to be explored with future prospective clinical studies.

It is also notable that even if the availability of a screening tool in referring centres increases the number of referrals into the system (due to changes in referring patterns by clinicians) or in line with the increase in demand seen across all ophthalmic services, the benefits of the screening step are still likely to be significant. Indeed, even a 25% increase in referrals into the system would still result in much shorter waiting times in the first few years and zero waits by year 6 in our service. Interestingly, although increasing the number of full laboratory-based EDT slots per week (increasing capacity) reduces the average queue time, when deploying the *RETeval*^®^ screening step, this effect seems to be dwarfed by the effect of the screening step.

With any screening tool, one concern is the effect of incorrect screening and this is the primary risk which needs to be addressed before clinical use of the device for the purpose studied here. Our simulation predicts that assuming a 95% threshold for both sensitivity and specificity, 107 (2.5%) of patients over the first 5 years would have an abnormal result at screening but a normal result when they have the full laboratory EDT (false positive screening). The potential impact that this would have on patients such as anxiety around a falsely abnormal clinical test, has not been studied here and would need to be explored before large-scale implementation of the *RETeval*^®^ ERG screening step. Indeed, it would need to be balanced against the fact that these patients would still have their lab-based EDT test and result significantly more quickly than they would have done in the current *status quo* model.

Most important is the concern around false negative screening. Our simulation predicts that assuming a 95% threshold for both sensitivity and specificity, 0.8% of patients would have a normal result at screening but would have been found to have an abnormal ERG result should they have had the full laboratory EDT as would be standard in the current *status quo* model. Additionally, with questions around the sensitivity/specificity, test/retest variability and useability in the clinical setting, the rate of false negative screening could vary in real-world practice. It is beyond the scope of this study to measure the impact of false positive and negative screening, and this would need to be explored safely in future implementation studies in a real-world setting.

In summary, our study has demonstrated that there could be significant benefits to patients and clinical services by adding a *RETeval*^®^ ERG screening step in the referral pathways for visual EDTs, should it prove to be useable, acceptable to patients and safe in real-world practice. We have shown that the DES modelling technique is a valuable tool in this early assessment and could also be utilised in future research on this topic. However, many questions remain about using the device in the real-world setting for this purpose. Our work supports the value of further prospective clinical studies seeking to optimise its useability, which patient groups it might be most useful for, test/retest variability, acceptability, sensitivity and specificity, resulting changes in clinical referral rates, the effect of the degree of clinician’s suspicion of an abnormal result and most importantly, the consequences of false positive and false negative screening.

## Limitations of the study

Patients referred for screening or monitoring (e.g. hydroxychloroquine screening or for Birdshot chorioretinopathy) were included in Group B for their first evaluation only and for subsequent appointments are not included in the screening model. These ‘special’ cases are therefore underestimated in terms of their burden to the overall backlog in this work as they are beyond the limitations of the current model.

Cases screened falsely as ‘normal; by the *RETeval*^®^ ERG (false negative screenings) are a very important group. We have looked here at the numbers and highlighted that this group need close attention in future prospective clinical studies but are unable to estimate the effect of this on the overall burden of repeated tests for cases where clinical suspicion remains high and retesting is requested ether with the *RETeval*^®^ or by lab-based electrodiagnostics.

In this study we have made some assumptions by grouping referral types into four categories (groups A-D). Within each group there are many different clinical presentations/clinical concerns/potential diagnoses due to the wide variety of patients requiring visual electrophysiology. So, it is likely that factors including the false negative screening rate and importantly, implications of a missed diagnosis will vary according to these various bespoke factors even within groups A-D. Further studies would be needed to dissect out whether some of these factors vary enough to mean that screening is suitable for some patient and not others, even within the broad clinical groups A-D.

For data collection, we used a 3-month window rather than a longer window due to the large amount of data. A longer window of data collection would be ideal in future studies.

This study is designed to represent a realtively top-level description of the potential effect of introducing a screening step in the clinical diagnostic pathway when factoring in the current real-world ‘unknowns’. The addition of more granular details into the model from future prospective studies, such as geographical data, real world data on test failure rates etc. would provide better insights into the nuances of the model and likely include a time series graph and other information about the performance of the model.

## Data Availability

The data that support the findings of this study are available from the corresponding author upon reasonable request.

## Appendix

**Table 4:** Parameter values

Parameter	Value	Source
Interarrival rate (base case)	0.333	Estimated based on retrospective data collection; exponential distribution
Fraction of arrivals in group A	0.395	Retrospective data collection
Fraction of arrivals in group B	0.072	Retrospective data collection
Fraction of arrivals in group C	0.205	Retrospective data collection
Fraction of arrivals in group D	0.328	Retrospective data collection
Fraction abnormal EDT group A	0.299	Retrospective data collection
Fraction abnormal EDT group B	0.286	Retrospective data collection
Fraction abnormal EDT group C	0.125	Retrospective data collection
EDT slots per day	12	Typical value for local service
Queue length for EDT at start up	228	Waiting list for local service 30.11.2019
